# “Family before Anyone Else”: A Qualitative Study on Family, Marginalization, and HIV among Hispanic or Latino/a/x Mexican Sexual Minority Males

**DOI:** 10.3390/ijerph19158899

**Published:** 2022-07-22

**Authors:** Moctezuma García, S. Raquel Ramos, Lisa Aponte-Soto, Tiarney D. Ritchwood, Laurie A. Drabble

**Affiliations:** 1 School of Social Work, San José State University, San Jose, CA 95112, USA; laurie.drabble@sjsu.edu; 2 School of Nursing, Yale University, Orange, CT 06477, USA; raquel.ramos@yale.edu; 3 College of Science and Health, DePaul University, Chicago, IL 60604, USA; lapontes@depaul.edu; 4 Department of Family Medicine and Community Health, Duke University, Durham, NC 27710, USA; tiarney.ritchwood@duke.edu

**Keywords:** HIV, sexual minorities, gay and bisexual, family, stigma, homophobia, Hispanic, Latino, people living with HIV, mother-son

## Abstract

This study explored the influence family relationships have on HIV-related factors among Hispanic or Latino/a/x Mexican sexual minority cisgender males in San Antonio, TX, US. A total of 15 young adults (7 people living with HIV; PLWH) ages 21–30 completed a semi-structured interview. Data were transcribed verbatim and analyzed using thematic analysis. The following themes emerged: (1) family support; (2) mother-son relationships; (3) father-son relationships; (4) sibling support; (5) family marginalization of sexual minorities; and (6) internalized homophobia. People who reported being HIV negative were more likely to have a prominent mother-son relationship, strong sense of family, supportive siblings, and family acceptance as a sexual minority. PLWH were more likely to report a weak sense of family, being raised in a maternal-led household, and less likely to have a relationship with their father and siblings. Marginalization among participants regardless of HIV status included exposure to religious rhetoric stigmatizing sexual minorities and fathers’ reinforcing Mexican traditional gender norms. In addition to encountering homophobia, PLWH were further marginalized by family members due to their HIV status. The findings suggest a need for greater attention to examining the impact of familial support of Hispanic or Latino/a/x Mexican sexual minority cisgender males as young adults with or at risk of HIV.

## 1. Introduction

Between 2008 and 2019, HIV diagnoses among Hispanic (descendants of Spanish-speaking nations) or Latino/a/x (descendants of Latin American countries and territories) men who have sex with men (MSM) increased by 18% within the United States (US) [1]. In 2019, Hispanic or Latino/a/x MSM accounted for 1811 new HIV diagnoses among individuals aged 25–29, 1439 among those aged 20–24, and 1382 among those aged 30–34 [2]. Texas has ranked number two in the nation of HIV incidence rates among Hispanic or Latino/a/x MSM [1]. The US government’s plan for ending the HIV epidemic has prioritized San Antonio, TX, US as an HIV-burdened area [3]; Bexar County in San Antonio has ranked third in Texas for the highest HIV incidence and prevalence rates [4]. In 2019, San Antonio had an HIV incidence of 14.0 per 100,000 compared to 11.1 for the nation [2]. Hispanics or Latino/a/x are less likely to be knowledgeable of their HIV status, achieve HIV viral suppression, and engage in pre-exposure prophylaxis (PrEP) [1,5]. A study conducted in San Antonio, TX, US indicated that Hispanic or Latino/a/x sexual minority males with a higher socioeconomic status (SES) were more likely to be aware of PrEP, but fears of being stigmatized as promiscuous discouraged them from enrolling in PrEP [6]. The same study found that Hispanic or Latino/a/x sexual minority males with a lower SES were less aware of PrEP and less likely to consider PrEP due to it being considered an experimental drug, medical side effects, and government mistrust [6]. Significant barriers attributed to racism, discrimination, and stigmatization have impeded efforts to mitigate HIV transmission among Hispanic or Latino/a/x sexual minority males [7,8,9]. Existing HIV prevention efforts to enhance access and engagement to HIV testing, HIV treatment as prevention, and PrEP have not tailored culturally informed interventions to address the needs of Hispanic or Latino/a/x sexual minority males [10,11,12]. Culturally informed preventive interventions that build trust in the community are needed to mitigate structural, social, and cultural barriers that increase the risk of HIV transmission among Hispanic or Latino/a/x sexual minority males.

Hispanic or Latino/a/x populations reinforce a collectivistic culture through familismo/familism, which encourages family members to prioritize the family instead of individual needs [13,14,15,16]. Research indicates that Hispanic or Latino/a/x male sexual minorities with a higher affinity for familismo are more likely to engage in healthy sexual behaviors that mitigate HIV acquisition [13,17]. Family acceptance and support play a crucial role in facilitating health-promoting behaviors (e.g., testing and health care treatment) and improving physical and mental health outcomes among young male sexual minorities who are HIV-positive [13,17]. Hispanic or Latino/a/x family involvement that reinforces cultural values may serve as a protective mechanism against risky sexual behavior among Hispanic or Latino/a/x male sexual minorities and strengthen supportive family systems [16,18,19,20]. Limited research is available on the implications of interpersonal family relations related to the stigmatization and marginalization of young adult Hispanic or Latino/a/x sexual minority cisgender males—males whose gender identity aligns with their sex assigned at birth. The aforementioned studies on familismo have focused on Hispanic or Latino/a/x sexual minorities ages 14–21 [19,20] and bisexual males ages 18–45 [14,17], but there is a gap in the literature exploring HIV-related factors associated with familismo among young adult (ages 21–30) Hispanic or Latino/a/x sexual minority cisgender males.

Despite experiences of acceptance and support, some Hispanic or Latino/a/x sexual minorities report feeling highly stigmatized by their families, religious community, and culture [16]. Hispanic or Latino/a/x sexual minority males may leave their home country or community to avoid being marginalized as a sexual minority and live more authentically as an openly gay or bisexual man [18,21] to protect their family from the shame and stigma of having a sexual minority son [22]. Religious beliefs may contribute to sexual minorities not disclosing their sexuality to others and increase risky sexual behavior, such as unprotected intercourse among male sexual minorities [13,23,24,25]. These experiences of interpersonal stigma are associated with adverse health and mental health outcomes. Hispanic or Latino/a/x male sexual minorities who experience rejection from their families are more likely to report severe physical and mental health consequences, which further compound the effects of racism and internalized homophobia [26]. Lack of acceptance was found to contribute to depression, substance use, and HIV risk [27,28]. Hispanic or Latino/a/x sexual minority males reported higher levels of negative family reactions to disclosure about their sexual orientation, as compared to their White counterparts [27]. When Hispanic or Latino/a/x sexual minority males were asked about their feelings regarding disclosure of their sexual orientation to their families, they expressed fear of isolation and rejection about disclosing their sexual identity and/or fear of violence from their families [22]. Additionally, Hispanic or Latino/a/x male sexual minorities reported experiencing violence at some point in their lives due to disclosing their sexual orientation. 

To date, limited research has explored the impact of family, marginalization, and HIV among young adult (ages 21–30) Hispanic or Latino/a/x sexual minority cisgender males, which is the age cohort at greatest risk of HIV transmission. Additionally, Hispanic or Latino/a/x sexual minorities in research are usually categorized as a monolithic ethnic group, which overlooks important cultural factors based on perceived nationality [29,30]. This study applied a qualitative methodology to further understand the experiences and perceptions of young adult Mexican sexual minority cisgender males related to interpersonal familial relationships to develop and enhance culturally informed HIV interventions. The purpose of this qualitative study was to explore what effect family members have on HIV-related risk and protective factors among young adult Mexican sexual minority cisgender males in San Antonio, TX, US. 

## 2. Materials and Methods

In-depth qualitative interviews were conducted with Hispanic or Latino/a/x Mexican sexual minority cisgender males. Purposive sampling was applied to ensure representation of participants with experiences relevant to the research questions [31], which for this study involved recruitment of participants both with and without HIV. The study was promoted in English and Spanish with recruitment flyers at gay venues throughout San Antonio, TX, US and social media outlets (Grindr, Facebook, Scruff). An emphasis was placed on San Antonio, TX, US because it has been prioritized as an HIV-burdened territory for ending the HIV epidemic [3] and there is a dearth of research exploring HIV-related factors among Mexican sexual minorities in San Antonio, which has more than 2 million residents that reported being from Mexico [32]. The study was also supported by the Alamo Area Resource Center (AARC, San Antonio, TX, US), an established local community-based HIV service provider. 

### 2.1. Procedures

Recruitment was promoted in person, online, and through AARC to strengthen communication efforts in English and Spanish to engage a more diverse sample for the study [33]. The principal investigator (PI; MG, author) also took into consideration positionality to assess their insider−outsider status to determine power dynamics based on multiple identities (e.g., race, ethnicity, sexuality, gender, sex assigned at birth) to engage with the community, recruit participants, and administer interviews for the study [34,35] with young adult Mexican sexual minority cisgender males. The PI made conscious efforts to take into regard subtle cultural nuances that may affect trust and rapport with the population of interest [34,35]. For example, the PI may be considered an insider due to their perceived racial or ethnic identity as a cisgender male, but may be considered an outsider due to their educational status or not being born and raised in San Antonio, TX, US—an outsider investigating their community. Efforts were made to enhance engagement and trust of the community by integrating “indigenous insiders” to promote the study, who are perceived as important members of the community based on their longstanding relationships in establishing trust, rapport, and support with local natives [34,35]. AARC case managers, HIV testers, and outreach staff played a critical role as “indigenous insiders” in recruiting and encouraging PLWH and their social networks to participate in the study. The PI met with staff, reviewed the consent form and research protocols, and encouraged staff to promote the study.

The PI screened potential participants for eligibility in their preferred language (i.e., English or Spanish) based on the following inclusion criteria: (1) identify as Mexican, (2) identify as cisgender male, (3) between the ages of 21–30, (4) reside in San Antonio, and (5) report a sexual encounter with another male in the past six months. The PI obtained consent from participants before proceeding with the interviews. Participants were informed that results would be published and any identifiable information would be removed to protect the privacy of the participant and their social networks. Participants received a USD $50 cash incentive for completing an audio-recorded interview for approximately 90 min. The local institutional review board assessed the study protocol to ensure participants were not exploited and incentives appropriately acknowledged participant contributions by compensating them for their time and travel. All interviews were administered at AARC and case managers offered their office space for the interviews, which provided a familiar environment for existing clients or raised awareness of HIV-related services for people not familiar with the agency. For example, some participants who were HIV negative were unaware of PrEP and after the interview they received resources on how to access PrEP at AARC or other local HIV service providers. 

### 2.2. Interview Guide

A semi-structured interview guide was used to explore the influence of family networks on the lives of Mexican sexual minority cisgender males in relationship to cultural beliefs associated with the following domains: family, well-being, support, sexuality, and HIV (Table 1). A semi-structured interview method was used to guide the participants in answering open-ended questions related to domains in an exploratory approach to encourage participants to share their experiential knowledge related to a particular social phenomenon [36]. For this study, it was important to explore if interpersonal familial interactions influenced HIV-related behaviors of Mexican sexual minority cisgender males as young adults. Semi-structured interviews allowed the researcher to ask general questions that required the interviewer to ask follow-up questions based on the participant’s response to gain a deeper understanding of their lived experience [36]. The PI administered and reviewed all of the qualitative interviews, which provided critical insight to determine data saturation by collecting sufficient data and determining that no additional insight had been provided by the previous participants [37]. 

### 2.3. Data Analysis 

Qualitative interviews were transcribed verbatim, analyzed in their original language, and names disclosed during the interview were removed from the transcripts. The PI applied Braun and Clarke’s [38,39] thematic framework by: (1) becoming familiar with the data; (2) generating initial codes; (3) searching for themes; (4) reviewing themes; (5) defining themes; and (6) summarizing research findings. NVivo software [40] was used to conduct a thematic analysis focused on identifying prominent themes based on codes associated with family. The PI interviewed all participants to ensure general questions were answered for each domain and followed up with probes to gain a deeper understanding on the implications of familial relationships for HIV-related factors among young adult Mexican sexual minority cisgender males. Additionally, the PI’s “insider−outsider” status was beneficial to the interview process. For example, some participants decided to complete the interview in English, but spoke Spanglish (mixture of English and Spanish) to express themselves. The PI’s insider status enhanced their ability to respond in Spanglish throughout the interview to enhance rapport and engagement with the participants, which provided greater insight into the participants’ experience [34]. The PI’s outsider status of not being a native of San Antonio, TX, US reinforced professional boundaries and avoided social conflicts as a community member [34], which increased objectivity for the researcher and reassurance of confidentiality for the participants. All interviews were administered, coded, and analyzed by the PI. 

An inductive analysis approach was applied to identify categories that emerged based on data from the participants’ responses [38,39]. The PI initially reviewed all transcripts to become familiar with the data and generated codes based on family roles (e.g., mother, father, sibling) and HIV-related factors. Thematic analysis commenced using a codebook and was refined iteratively, and previous transcripts were recoded to enhance reliability and application of codes from the refined codebook [39]. A semantic approach was applied to generate themes based on participants’ statements [38]. Thematic analysis for this study was further guided by an essentialist/realist paradigm, which is consistent with an inductive approach and amplified participants’ experiential knowledge based on the social phenomenon [38]. NVivo software [40] was used to highlight prominent codes and identify emerging themes for the entire dataset. Multiple steps were taken to limit research bias, which included utilizing a refined codebook for analyzing the data, identifying quotes to support themes, and reviewing emerging themes with co-authors [39]. Analysis also explored differences and similarities within themes between the narratives of participants with and without HIV. The themes were further reviewed to ensure they were supported by the coded extracts and all of the transcripts [38]. Although an existential/realist paradigm was employed throughout the data analysis, authors’ existing knowledge of the literature and theoretical perspectives did have influence in providing structure and addressing the research question, “What influence do interpersonal familial relationships have on HIV-related factors for young adult Mexican sexual minority cisgender males?” Direct quotes when necessary were translated from Spanish to English by the PI.

## 3. Results

A total of 15 participants (7 PLWH) completed semi-structured interviews in English. The majority of the participants identified as gay (93%) and reported being single (73%) at the time of the interview. The annual household income of participants was less than USD $39,999 (87%) and approximately half of the participants reported having some college education or higher. Table 2 provides detailed participant demographic information.

### 3.1. Themes

The following themes emerged from the data analysis: (1) family support; (2) mother-son relationships; (3) father-son relationships; (4) sibling support; (5) family marginalization of sexual minorities; and (6) internalized homophobia. Table 3 provides a summary of designated themes and subthemes for the study. Pseudonyms were assigned to each participant and were used for direct quotes throughout the results section to protect the identity of participants.

#### 3.1.1. Family Support: “Family before Anyone Else” 

Descriptions of family support were important in the narratives of all participants, although dynamics typically varied by HIV status. The majority of the participants without HIV indicated that they had a strong supportive immediate and extended family network. For example, Rodrigo, a participant, explained: *“At the end of the day, family is all you have and my family is closer than most families so I cherish it way more—I always put my family before anyone else.”* Other participants elaborated about specific ways they experienced familial support, exemplified by Emiliano: *“My grandfather, he’s always been like another father to me. I can call him at any time of the day or any time at night and talk to him about the littlest problem that I’ll have...”* Similarly, Emilio noted that: “*My immediate family and my extended family such as my grandparents or my aunt, we are all very close together.*” He also commented on how his family stayed connected, *“Because we have our Sunday dinners, always see each other regularly throughout the week, we’ve always been very close and we’ve always had like an open communication with each other.”*

Unlike their peers, most PLWH reported familial isolation or limited familial support. Alejandro described how being raised by multiple family members had resulted in a tenuous sense of family support and relationship with his parents as a young adult:

*I wasn’t raised by my* [parents]… *I’m the youngest of four children. I had a brother who passed away when I was young, but I was born out of wedlock at a time when my parents were splitting up, so as a result, I wasn’t raised by my parents until about the age of five. During that time, I was raised by multiple family members and extended family, and so I understand that I have probably some resentment towards my parents for that. I think that is part of that strenuous relationship.*

Some participants described being marginalized due to their sexuality. Matias emphasized that he is the “*black sheep of the family*” and Sebastian indicated that *“…they* [immediate family] *don’t hate me being gay, but they’re not okay with it.*” However, PLWH were more likely to rely on fictive kin for support. Leonardo indicated that “*I don’t have a family right now”* but referred to his best friends as sisters because they accepted him unconditionally when he was kicked out of his house at the age of 15 for being gay: “*They were all about supporting me and loving me… I always have a place with them to live.*” 

#### 3.1.2. Mother-Son Relationships: “Mother Is Always Right”

The majority of participants without HIV identified their mothers as a critical source of support, which influenced their decisions to engage in safer sexual practices. This was typified by statements from Diego, who explained: “*I always listen to what she says, they always say mother is always right and she told me to always use condoms and I always have.”* Gael noted: *“Oh, yes, my mother tells me, ‘Go get tested just to be on the safe side; it’s always great to catch it* [HIV] *early if you do get it or whatever’.”* Some participants described how their mother influenced them to modify risk during particularly stressful times. For example, Rodrigo observed that “*after every bad relationship I go out there and get kind of crazy”* and although both parents were supportive, *“my mom’s always the one to sit me down and tell me I need to be careful. I need to make sure I’m protecting myself—she doesn’t want anything bad to happen to me.*”

Some of the descriptions of maternal influence were sometimes less direct, but reminders to wear condoms were still perceived as impactful and caring. For example, Jesus explained: *“It’s funny because… we didn’t talk intimately like that growing up, but I guess she* [mom] *see’s that I’m happy and that I like men. She talks to me about it, ‘Nomás ten cuidado, usa condones’* [Just take care, use condoms]*.”* Similarly, Daniel observed: *“The random mention of the condom—as weird as it sounds, it does help because I know that they’re* [referring to his mother] *looking for my best interest, and something like that.”*

On the other hand, more PLWH reported a weak or strained relationship with their mother. For example, Sebastian characterized his relationship with his mother as “*kind of shaky”,* noting that “*I guess we’ve never been able to talk about anything.”*

#### 3.1.3. Father-Son Relationships: “Buck Up, Kiddo.”

Most of the participants without HIV emphasized that their fathers were instrumental in providing tangible support, but no emotional support; exemplified by a description from Leonel:

*For my father, him being a parent was that he bought a house, paid for our clothes or shoes, made sure that we ate, but that was it. He never showed no emotion or support towards us. So, my dad thinks that he did a great job because he did all that, but to me personally I don’t think that he did a great job. I feel that there is more to being a parent than just buying the necessities. You also have to provide the support, the emotional support and reassure your child that you love them or that you’re there for them. I didn’t get that.*

Other participants described having a constrained father-son relationship. Daniel explained:

*With my dad, it’s the same way, but he’s more, I guess, reserved in his emotional aspect. I mean I can still come to him but he’s more reserved. Well, he kind of… I guess,* [has] *machismo. My dad’s army, my dad eventually got around to joining the military. So military values were definitely instilled in my dad’s heart, so everything is like, “Buck up, kiddo*.”

The majority of PLWH were more likely to report not having a father figure or a weak relationship with their father. Some participants described these weak connections in the context of growing up with family dysfunction or violence, such as Leonardo:

*Yeah, a lot of physical fighting. Then he* [father] *would leave, and then she* [mother] *would forgive him and he would come back. It was off and on like that, and I really hated him for it because I would always try to jump in and hit him while he was trying to hit my mom. I was trying to protect my mom… He would tell me, “Fuck you.” He would cuss me out and he would leave. Then the next day, she’s there hugging him and I would have this anger towards my mom already, like, “How could you go back with him?”*

Other PLWH described their fathers as being absent. For example, Emilio explained that his father “*was in prison most of my life, he had been in and out of jail*” and that “*he is my father, he is my family, but I really never had any interaction…*”. He elaborated on ways he and his family were disconnected from his father: *“My mom left him. When we did see him, we were visiting him. We weren’t visiting as an entire family. It was either my sisters and I with my dad, or my sisters and my brother with my dad. It was never my dad with our family.”*

#### 3.1.4. Sibling Support: “Love Me No Matter What.”

The majority of the participants described social support and relationships with their siblings as an important part of their lives, but it was typically more current and robust among participants without HIV. Some participants without HIV described that their siblings provided a critical source of support for them when they encountered issues with their parents. For example, Daniel described the profound positive impact of his relationship with his brother:

*He* [participant’s brother] *just told me that he’ll love me no matter what, which is awesome. I cried* [laughs]. *He told me that he’ll love me no matter what and that he’ll always be there for me and if I ever have any problems I can always go to him regarding anything and that was wonderful because I love my brother so much, he means the world to me.*

Similarly, other participants described deep and enduring support from siblings, typified by the following story from Leonel:

*I was 14 still. I came out to my sister first and it was great. That’s when me and her got so much better. Because she told me that she knew what I was, and she hated that I denied it, so she would try and get me to come out, but I didn’t. So, she hated that part from me, and so when I did come out, she was happy for me, so she was there for me. My brothers, they said they accepted it at first, but they were very still enclosed from it. They said it, but they didn’t show it that they were. It took them a while, but within a couple of years, they also came around, and now we have the great relationship that we have now. Because when I did come out, they went against my parents because they supported me. So, our family was divided. My parents didn’t talk to us, and they didn’t talk to them for a good while, because they supported me.*

Jesus also recalled an incident where his brother “*lashed at Dad about homophobia*.” When Jesus asked his brother why he spoke up, his brother told him *“…that he already knew I was* [gay] *and he just was trying to protect me, so maybe in the future Dad will not be as homophobic or not be homophobic, but he did that just because it was a sense of protection because of me.*” He described what his brother’s support meant to him, explaining, “*When I heard that, I started crying, just because I know that I can count on my brother for everything—I can tell him everything, and like I said, to this day, we’re best friends.”*

Conversely, PLWH often described a distant relationship with their siblings and feeling a sense of isolation from the family. In some cases, the process evolved over time, with some variation in how that distancing was perceived as a loss. For example, Mateo described that he used to hang out with his little brother and a close friend*, “but now times have changed and everyone has their own lives. My little brother has his family, homeboy has his girlfriend, and I’m just chilling by myself, but whatever, I don’t care.”* Similarly, Leonardo observed that he *“used to laugh and joke about things”* with his siblings, but now *“they don’t have time”* and *”they don’t joke around anymore—it makes me feel like I lost my brothers and sisters.”*

#### 3.1.5. Family Marginalization of Sexual Minorities: “There’s a Set of Traditions You Kind of Have to Follow.”

All participants regardless of HIV status described that their sexuality was a critical source of feeling marginalized by family members due to cultural factors reinforcing heteronormative religious and gender ideals. Religion was emphasized as being ingrained in Hispanic or Latino/a/x culture regardless of a participant’s religious involvement as an adult. Daniel shared his experience on the significant role religion has played in reinforcing family values:

*Yeah, because that is another aspect that kind of goes in line with family values and how you’re brought up. It’s no coincidence that the majority of Hispanics and Latinos, their religion is Catholicism. So, that kind of happens how you grow up, because if you’re Hispanic or Latino your kind of, I don’t want to say expected to be Catholic, but* [chuckles] *you got to grow up in those values, and the same thing with your family, there’s a set of traditions that you kind of have to follow.*

Mothers and grandmothers were more likely to be religious and marginalize sexual minority family members. Although some participants described this dynamic changing over time, typified by the following observation from Santiago:

*I feel it is because it’s always… typical Catholic mother. It’s a Mexican Catholic mom. She’s always praying, and always lighting candles, and always going to church, and to this day, I don’t really understand how my mom can pray every single day on the hour, every hour. That’s not what she was taught and that’s not what the Bible tells you. She specifically told me that too, and now that years have passed by, she tells me, “I realized then I wasn’t thinking right. My mind wasn’t open to the fact that I have a gay son and that it’s fine and there’s nothing wrong with that.” It’s taken her a while.*

Dealing with stigmatizing religious messages was frequently described in narratives about struggles with sexual identity, self-acceptance, and coming out, as Leonel indicates:

*Well, a lot of my family’s very religious. Very religious actually. So, religion did play a good part in it. It even still played a part when I came out. For a while my parents sent me to church. They took me to church. I had to go to church, and I did. I went to church. It was nothing though. It didn’t do anything. At that time, I was already assured. I was already like, “You know what? I’m gay.” Yeah, I wasn’t too fully understanding of everything because it was in high school. So that time I came out, I wasn’t too fully understanding of what it was to be gay, or who I was going to be, how I was going to be, but I knew that I was already like, I’m gay, though. This isn’t going to do anything to change it. Maybe it will strengthen my relationship with God. Because I don’t believe that He hated me or that He didn’t want me. As I had earlier said that I almost died inside my mom and I always think back to myself and say, “Well, why didn’t I?” I always think that maybe He did want me here.*

Marginalization of sexual minority children was distinct based on the gender of the family member. Religious beliefs were more likely to influence female family members, but male family members were more concerned about idealizing what it meant to be a Mexican masculine male.

Most of the participants expressed that their sexuality caused their fathers to perceive them as less of a man. Daniel explained: *“I was raised to believe that if you’re a Mexican or Hispanic male, you again had to act a certain way.”* Daniel further elucidated that family expectations of being a Mexican male were further complicated due to the intersectionality of religiosity, sexuality, and gender, noting: “*One way was, unfortunately, you’re not really supposed to be gay. You’re supposed to be macho, and provide for your family, and work hard and carry on, like, be Catholic I guess, too.”* Violating gender expectations was connected to paternal rejection for some participants. Diego explained that he was kicked out of the house as an adolescent when he came out as gay to his father*; “He told me, I’m not your son, you’re a mistake. Telling me all this stuff, and I guess he didn’t like it—the fact of me being gay, so he just left me with some random people.”*

For others, paternal messaging around gender norms took the form of bullying, teasing, or “macho-like” behavior. Rodrigo described learning to protect himself against bullying from his father:

*My dad has always been kind of like a bully, I guess. He’s always made fun of people, and even to this day he still does. That’s just his way. He knows that. He accepts gay people and he’s had my friends in his house and stuff, but he always just for some reason still makes fun of them, and sometimes he’ll make fun of me because I’m gay, but I’ll just make fun of him in a way. He calls me his daughter. He says I’m his daughter, I’m not his son. That’s him just making fun of it. It doesn’t bother me.*

PLWH encountered family members that stigmatized them for being a sexual minority, but their HIV status caused family members to physically distance themselves or further stigmatize them. For example, Leonardo explained:

*I think that they don’t care* [about me being diagnosed with HIV]. *It’s not that they don’t care, like I said, I don’t think they understand it. In the past when I’ve gone over, one of my sisters has not let my nephew drink out of my cup. She doesn’t know—it hurt my feelings but I didn’t say anything about it, I just brushed it off. That’s pretty much about it. As far as hugging and hugging the kids, and hugging my brothers and sisters, they still allow me to do all that.*

Similarly, Matias described how he was marginalized by his brother when he disclosed his HIV status:

*Basically, we weren’t talking anymore and then when he* [participant’s brother] *found out* [I was diagnosed with HIV] *he was just like—he wasn’t living with us anymore he was like, “I still can’t believe you’re letting him live there. You’re going to risk* [refers to his sister by name].*” He was like, “You’re going to risk her life.” I was just like, “It’s not contagious like that. It’s only contagious sexually.” “Basically, if I hug you or touch your hand,” I was like, “you’re not going to get infected.” I was just like it happened sexually and I was like, “You have to have sex with someone in order to give it to someone?”, and I was like, “If you don’t give it to them then probably that’s just a lucky person then basically.” Yeah, he hates me more because of that.*

Some participants also described how family members blamed their sexuality for their HIV status. For example, Emiliano described challenging his father’s stigmatizing attitudes about his sexuality and health:

*He was saying, “This is why I didn’t approve of your lifestyle,” this and that. Pretty much that’s how the conversation went. I told him, “Well, Dad, how much more different if I was straight? If I banged some random chick behind the club, and then nine months later, she’s pregnant and I might have something, or a combination of both.” I told him, I said, “At least, thank god and goddesses, at least I got one thing I had to deal with.”*

#### 3.1.6. Internalized Homophobia: “I Should Have Kept My Mouth Shut.”

Many participants went through a period of internalized homophobia based on their experiences of being marginalized by family members for being a sexual minority. Some questioned how their life would have been different if they were straight. Sebastian wondered: “*I don’t know what it is, but if I could, I would like to be straight and just have a normal life.”* He went on to clarify: “*I’m not saying that that’s a normal life because being gay is normal too, but for me, the way I grew up, I view that as normal.”*

Others commented on struggling with disclosure to family or issues they encountered with family members in becoming a young adult sexual minority, as reflected by Leonardo:

*It was scary, but at the same time—sometimes I still struggle with it* [being gay]. *Sometimes when I see my life... when I’m not happy, or something, or when I’m depressed, I feel like I should have just kept my mouth shut and not told anybody I was gay. Because most of my cousins are all in the Navy, or in the service, and there’s no other gay person in my family. I was thinking maybe I should have just stayed quiet and not said anything, joined the service and moved on, and been good.*

Most participants shared how their sexuality conflicted with familial and cultural expectations, which manifested in internalized homophobia. Participants constructed new meanings and “ideals” about being a Mexican sexual minority cisgender male, exemplified by Daniel, who explained:

*…if you’re short of the ideal of course you’re going to be questioning yourself like, “Well, why am I this way, what am I lacking, and am I even lacking?” However, you kind of look at it and analyze like, well why is their ideal this way, and why can’t I be my ideal, but eventually …through life experience you can make up your own decision, and you can, you know, make your own ideals on what it means to be Hispanic and gay and etcetera.*

## 4. Discussion

The purpose of this qualitative study was to explore the role of family members in relation to HIV risk and protective factors among young adult Mexican sexual minority cisgender males in San Antonio, TX, US. This study has contributed to the literature by describing differences in family support dynamics between young adult sons who are HIV positive and sons who are HIV negative. Six themes emerged (1) family support; (2) mother-son relationships; (3) father-son relationships; (4) sibling support; (5) family marginalization of sexual minorities; and (6) internalized homophobia. Our findings suggest that family support provides a critical role in mitigating HIV-related risks for young adult Mexican sexual minority cisgender males who reported not having HIV, but narratives of PLWH were generally characterized by more strain and disconnection with their families. However, this study found that fictive kin played an important supportive role for PLWH shunned by their biological family, which reinforces other research findings related to protective factors associated with fictive kin for the Hispanic or Latino/a/x community [41].

In the current study, participants who identified as being HIV negative had strong relationships with their mothers. Specifically, mothers that were more supportive and nurturing of their sexual minority sons encouraged them to engage in protected sex. Our findings corroborated another study’s findings that parental bonding and social support were negatively associated with PrEP stigma and positively associated with engagement in health care [42]. Maternal bonding in particular played a significant role in family engagement in health care [42]. This highlights how matriarchy in Hispanic or Latino/a/x families is instrumental in influencing overall family engagement in health promoting behaviors, such as health care engagement and HIV prevention behaviors in young adult sexual minority men. Other similar studies examining the influence of parental support on HIV prevention behaviors found that PrEP interest and use was low in sexual minority adolescents who did not have existing parental support [43], as well as HIV testing [44]. PLWH in our study described more strain in relationships with mothers and rarely described support that influenced health behaviors. This study corroborated other research findings on the negative implications (internalized and externalized homophobia) of religious beliefs among Hispanic or Latino/a/x sexual minorities [16]. Regardless of HIV status, participants in this study that referred to their mothers and grandmothers as religious were more likely to experience being marginalized due to their sexuality. 

In the current study, fathers were overall described as very withdrawn from their sons’ lives, which was particularly evident among PLWH. Other than providing the necessities of life, fathers remained culturally aligned with masculinity and emotionally withdrawn from their sons lives due to their sexual identity and HIV status. Participants that reported being HIV negative in this study were more likely to have a father-son relationship, but experienced being ridiculed as being less of a man due to their sexuality. Although fathers met the physiological needs of their sons that reported being HIV negative in this study, persistent messaging on heterosexual masculinity ideals may lead to internalized homophobia and/or sexual orientation concealment [45] and deteriorate the positive implications that stem from their father as a provider. PLWH for this study were more likely to report that their fathers did not provide tangible support in addition to being emotionally unavailable, which limited access to resources and increased health risks. Discordant dynamics, such as this, may impact mental health and subsequently increase HIV-related risk behaviors. 

Our findings highlighted sibling support was important to the health and well-being of study participants, but quality of supportive sibling relationships was generally weaker in the narratives of PLWH. Narratives suggested that unsupportive siblings, as well as other family members, had low knowledge about HIV acquisition. This misinformation fostered physical and emotional distancing in familial relationships. Lack of sibling support heightened perceived stigma associated with HIV and internalized homophobia. However, participants that reported an HIV negative status were more likely to identify their siblings as an important source of support during the coming out process and addressing homophobic attitudes within the family, which became an important protective factor for young adult Mexican sexual minority cisgender males. Facilitating increased familial support is important; other research has suggested that such support is protective and decreases HIV risk for sexual minority males [42,44]. Interventions targeting heterosexual Mexican siblings and fathers of sexual minority men are needed to reframe masculinity, increase awareness of how HIV is contracted, and strengthen familial bonding. Interventions of this type are critical to dismantling repeated cycles of cultural gendered masculinity in Mexican men who have a sexual minority son or sibling. 

This study has a few limitations. First, our sample size was small and findings cannot be generalized to a larger population. Although small, our findings align with qualitative sample sizes from similar literature. Our findings also align with the literature on the role of family support for sexual minority men. Second, our sample was comprised of Mexican sexual minority cisgender males, which also decreases generalizability to Hispanic or Latino/a/x sexual minorities. However, since Hispanic or Latino/a/x identity is not monolithic, our findings are promising to further understand cultural factors associated with HIV among young adult Mexican sexual minority cisgender males. Third, qualitative research does not leverage quantitative outcomes. We utilized Braun and Clarke’s [38,39] thematic framework and applied the methods of rigor to this qualitative study. Although not quantifiable, our qualitative findings provide substantial context for a larger mixed methods study to further examine the role of family support in young adult Mexican sexual minority cisgender males. 

Despite these limitations, this study revealed important insights about ways that Mexican sexual minority cisgender males benefit from family support as young adults regardless of HIV status. Maternal support is an important protective factor that mitigates exposure to HIV by enhancing the well-being of sexual minority children as young adults. Research findings also emphasize the importance of sibling support during the coming out process to mitigate negative reactions from parents and moral support as young adults. This study further highlights potential mental health issues, such as internalized homophobia, that can ensue from limited or withdrawn parental and sibling support for PLWH. The significant role of masculinity in Mexican culture may increase HIV risk and/or lead to mental health issues due to lack of unconditional affirmation regardless of sexual identity and HIV status. A qualitative study explored the HIV care continuum in Hispanic or Latino/a/x sexual minority men and found that cultural masculinity resulted in avoidance of HIV prevention strategies in order to avert being stigmatized as non-masculine [46]. More research is needed in this area with a focus on interventions that center intersectionality, relationship building, and trust between sexual minority sons and their siblings and parents. Additionally, interventions should respectfully dismantle culturally relevant ideals of masculinity that depict sexual minority men as less-than or threats to the traditional family paradigm. 

## 5. Conclusions

The findings of this study underscore the potential impact of interventions that may facilitate familial support of young adult Mexican sexual minority cisgender males, including PLWH. Specific foci for interventions may include strategies for strengthening family bonding, increasing awareness of how HIV is contracted, and reframing masculinity for fathers and male siblings. Findings also point to the importance of coping with interpersonal stigma, including stigma based on religious beliefs, and fostering social support among Mexican sexual minority cisgender males, particularly PLWH. Additional research on culturally responsive HIV prevention strategies appropriate for Mexican sexual minorities are warranted and interventions that reduce stigma and increase support within family networks may be one fruitful area for investigation. 

## Figures and Tables

**Table 1:** Semi-structured interview questions exploring the influence that family had on the well-being of Mexican sexual minority cisgender males ages 21–30 in San Antonio, TX, US.

Domain	Sample Questions
Family	Can we start by you describing your immediate family (specify members in the family, influence they have on your life)—remember not to use names, just their role (e.g., mom, dad, brother)?
	What influence has your family (specify who) played in helping you become who you are and how?
Sexuality	What role does your sexuality have on how your family perceives you?
	Who would be the best person in your family to talk about issues related to sex in general? Explain why.
	Would you feel comfortable telling your family that you are gay or bisexual? (Specify who they would tell and how the person would react.)
Substance Use	Explain how your family played a role in using or not using drug (probe for the following: alcohol, tobacco, marijuana, cocaine).
	Who would be the best person in your family to talk to about drug use? Explain why.
Religion/Spirituality	How does your family reinforce your religious/spiritual beliefs or values?
	Which family member would you say plays a significant role in your religious/spiritual beliefs?
HIV	[FOR PEOPLE WHO ARE HIV NEGATIVE]: Would you feel comfortable telling your HIV status to a family member if you recently found out that you were HIV positive? (Specify who they would tell and how the person would react.)
	[FOR PLWH]: Have you told a family member that you have been infected with HIV? If so, please share your story the first time you told a family member and what happened? If not, please explain why not and what are some of your concerns?
Health/Well-being	What role does your family play in encouraging you to stay healthy and how? (Specify family members.)

**Table 2:** Participant demographics of Mexican cisgender males (Mean age = 24.8) in San Antonio, TX, US.

Characteristic	n
HIV Status	
Negative	8
Positive	7
Sexual Identity	
Gay	14
Bisexual	1
Relationship Status	
Single	11
Partner/Boyfriend	3
Separated	1
Annual Household Income	
$10,000–19,999	5
$20,000–29,999	4
$30,000–39,999	4
$40,000–49,999	1
$60,000–69,999	1
Education	
HS or Less (No Diploma)	3
HS Diploma	2
Professional Trade	1
Some College	7
Bachelor’s Degree	1
Master’s Degree	1

**Table 3:** Themes and subtheme descriptions related to family, marginalization, and HIV status.

Themes	Subthemes among People Who Were HIV Negative	Subthemes among PLWH
Family Support: “Family before anyone else”	Described family as a reliable source of support, sense of familial belonging.	Described limited or inconsistent support by biological family members, but relied on fictive kin for support.
Mother-Son Relationships: “Mother is always right”	Described a nurturing mother-son relationship, including support that strengthened HIV protective factors as young adults.	Described weak mother-son relationship throughout childhood and strained relationship in young adulthood.
Father-Son Relationships: “Buck up, kiddo.”	Indicated that the father-son relationship provided tangible support throughout their lives.	Described their father as absent throughout their lives or recalled childhood encounters with their father as violent or strained.
Sibling Support: “Love me no matter what.”	Reported strong relationships with siblings that provided an important source of support; particularly during the coming out process and addressing homophobia within the family.	Reported distant or inconsistent supportive relationships with siblings, which created a sense of loss and isolation from the family.
Family Marginalization of Sexual Minorities: “There’s a set of traditions you kind of have to follow.”	Experienced marginalization from their mother and/or grandmother reinforcing religious homophobic rhetoric. Experienced marginalization from their father reinforcing masculine ideals and stigmatizing their sexual minority son for not meeting expectations of being a Mexican masculine male.	Experienced isolation and discrimination from family due to their HIV status, in addition to marginalization as a sexual minority.
Internalized Homophobia: “I should have kept my mouth shut.”	All participants regardless of HIV status experienced at some point throughout their life dealing with internalized homophobia and feeling marginalized due to their sexuality by family members. Participants expressed going through a critical stage of self-exploration based on the intersectionality of ethnicity, family, gender, and sexuality as a Mexican sexual minority cisgender male.	

## Data Availability

The data presented in this study are available on request from the corresponding author. The data are not publicly available due to potentially identifiable information and protecting the anonymity of the participants.
